# “At this age, a Moroccan woman’s life’s work is over”-older Moroccan-Dutch migrant women’s perceptions of health and lifestyle, with a focus on Ramadan experiences: qualitative research integrating education and consultation

**DOI:** 10.1186/s12939-020-1141-9

**Published:** 2020-03-14

**Authors:** Karlijn Koudstaal, Petra Verdonk, Edien Bartels

**Affiliations:** 1 Nutricentrum, Almere, The Netherlands; 2 Amsterdam UMC-VUmc, dept. Medical Humanities, Amsterdam Public Health research institute, Amsterdam, The Netherlands; 3 VU University, Dept. of Social and Cultural Anthropology, Amsterdam, The Netherlands

**Keywords:** Health, Lifestyle, Older Moroccan-Dutch migrant women, Qualitative research, Intersectionality, Ramadan

## Abstract

**Background:**

Older Moroccan-Dutch migrant women exhibit high rates of diabetes, hypertension, overweight and obesity which is further compounded by their high risk of multi-morbidity. Healthcare professionals’ efforts to encourage this group to adopt a healthier lifestyle have little success. We ask ourselves whether the concepts used in health education and promotion relate to these women’s experiences and beliefs. Today’s pluralistic Dutch society requires a more differentiated and applied approach, not in an essentialist way but in awareness that translation of rather individualized concepts like health and lifestyle is not always adequate, as the meaning and interpretation of such concepts may differ and may be related to women’s other (fundamental) perceptions. This can have practical consequences for health promotion and education. The aim of this explorative, qualitative research, conducted between April and September 2015 and taking an intersectional approach, was to explore older Moroccan-Dutch women’s perceptions of health and lifestyle and to analyse these in a broader context, related to other fundamental forms of identity such as gender, culture and religion.

**Methods:**

We recruited women with Moroccan backgrounds by approaching Moroccan women’s organisations and using the snowballing method (chain-referral sampling). Seven ‘natural’ group discussions were held (amongst women who regularly meet each other, aged between 22 and 69 years), and twelve in-depth interviews and an observation day (with women from 40 to 66 years). The transcripts were then analysed using thematic content analysis.

**Results:**

Five major themes were identified. Health was perceived of in the terms used in prevailing health promotion discourses in the Netherlands, but lifestyle was interpreted in a much broader sense than the current health promotion debate allows; it is not seen as an individual responsibility or as something an individual could control on their own, and the social benefits of health behaviours appear to outweigh the health benefits themselves. Lifestyle was located in three main social identities of the women: Moroccan, Muslim and mother. Finally, Ramadan played a huge and dominant role in the lifestyle experience of older Moroccan women and was central in this research.

**Conclusions:**

The finding that lifestyle is not seen as an individual responsibility but is located in social identities, can be applied to other settings that older migrant-Dutch women occupy. Further research will clarify this.

## Background

In the Netherlands, the highest prevalence of chronic non-communicable diseases (NCD) is found in migrant groups, especially in the first generation [1] [2]. The most important risk factor for all migrant groups, and especially for older people within these groups, is hypertension [3]. In the Netherlands, there is a difference in the prevalence of NCD between migrant men and women, with a higher prevalence of diabetes and hypertension as risk factors for cardiovascular diseases in migrant women, especially Moroccan-Dutch women. This group also has a higher risk of developing multi-morbidity than their equivalent male counterparts and migrant Turkish-Dutch older women [4]. Cultural aspects of lifestyle are seen as an important cause of this problem [5].

Researchers indicate that migrants with chronic diseases should be counselled about lifestyle improvements to prevent chronic diseases [4]. However, encouraging people to be more physically active and to adopt a healthy diet proves to be more difficult than just providing information. Programmes and educational courses have limited impact on the general public and even more so on migrant groups and people of lower economic status (SES) [6]. Health messages, interventions and educational programmes are often ill-matched with migrants’ perceptions and beliefs about health and lifestyle. In the Netherlands, for instance, many older people with a Turkish or Moroccan migrant background assume that ‘taking rest’ rather than being active will reduce symptoms, and taking part in sports is often seen as ‘typically Dutch’ behaviour. Physical activity is frequently limited to walking to the neighbourhood mosque and running errands [7]. Changed eating patterns after migration to the Netherlands, such as irregular eating during the day and skipping breakfast also contribute to the development of risk factors related to chronic disease such as overweight.

The social meaning of meals and food may also play a role, illustrated clearly by the abundant amounts of food served as a symbol of hospitality when receiving guests [8]. Hence, Turks and Moroccans’ cultural beliefs and practices may be one of the barriers to following healthy lifestyle advice [9]. When these lifestyle behaviours are so difficult to change, the question arises: how are cultural beliefs and practices related to each other and to other (fundamental) perceptions? What do health professionals need to know to be able to understand their Moroccan-Dutch women clients and develop a more successful approach? The concepts ‘health’ and ‘healthy lifestyle’ are used by health professionals. The understanding of health behaviour in Western society has come to be associated with individualistic ideas which emphasise the individual’s responsibility for health. Simultaneously, health has become a major pursuit in our societies [10]. According to Crawford [11], in Western society, ‘health has become a focal *signifying* practice’ or a ‘key word’. Although health is interpreted in many different ways (both biomedically and psychosocially), healthy eating and exercise play a dominant role in the discourse around health and health promotion. These concepts are hierarchically associated: health has priority and can be retained and promoted through lifestyle. Currently, lifestyle has even become a form of ‘moralised persuasion’ which divides society into two groups - those citizens who conform and those who deviate from health-related moral norms [12]. Older Moroccan-Dutch women are not only known for their high prevalence of NCDs, but also as a group which deviates from these health-related moral norms and is overweight or (often) obese.

This research starts from the perspective of those whose lifestyles are subject to the research. The focus is on their ideas, thinking and behaviour, not only in relation to food and exercise, but to health and daily life in general. Not all groups conceptualise an individual meaning of lifestyle, physical activity and nutrition. Some see lifestyle, not as a determinant of health, but more as a social (collective) activity [6, 13]. This different understanding creates other, new, opportunities for health-promoting interventions. Current interventions are predominantly based on individualistic psychological and biomedical concepts and are less orientated toward health and well-being as social phenomena. When health is seen as a more social phenomenon, it can be related to identity, age, gender, religion, ritual and views on the body; all highly socially embedded concepts. The aim of this qualitative study is to explore what views Moroccan-Dutch migrant women above the age of 40 years have on lifestyle and its relationship with health. The findings will provide insight into the women’s needs and illustrate how these needs can be addressed in a culturally appropriate way.

## Methods

Societal factors such as migration, transnationality, age and gender, culture and ethnicity, religion, socio-economic position, health literacy, marginality and their interactions play a role in the health and lifestyles of these women. Besides the Dutch context, the family in the country of origin is important too. Daily lives are mixtures of Dutch and Moroccan ethnic and religious styles and complex interactions of many social and economic factors, which are also related to health outcomes. The women’s health and lifestyle activities are linked to, and interact with, their social identities. As the women’s identities are shaped by gender roles and other aspects of their identity such as religion, cultural practices, and age, we used an intersectional framework of multiple intersecting categories of difference and identity [14].

The supposed differences in perceptions on lifestyle, on the one side an individualistic concept in which individual responsibility for health is central, and on the other side a social phenomenon bound to collective activity for women with a migrant background, makes a cognitive based model like the Theory of Reasoned Action (TRA), the Theory of Planned Behaviour (TPB) and the Trans-Theoretical Model (TTM) too limited. That’s why we need a model in which social variables are included. The Health Belief Model includes demographic and socioeconomic variables and is a potentially effective tool for elaborating more variables (for example on intersectionality) [15]. Unfortunately in practice, the HBM model has not often been used effectively to exploit this potential strength [16]. Besides, ‘just’ taking social variables into account is not sufficient. To understand the concept of lifestyle as social phenomenon requires a model which includes communal identities. Social phenomena are relevant in one’s own group but also in negotiation with other groups, a process which is typical in today’s pluralistic society.

**Fig. 1:**
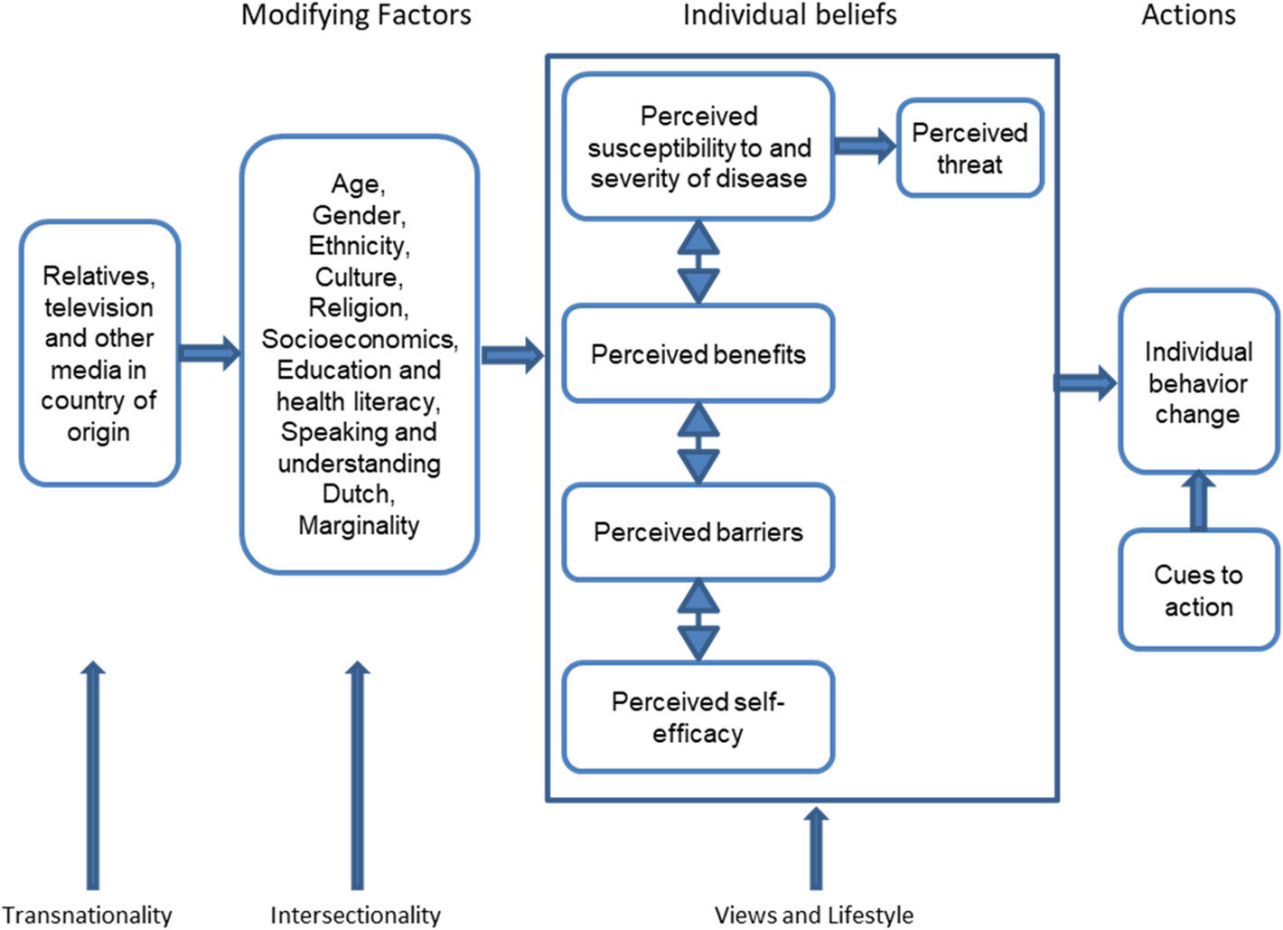
The Health Belief Model (based on Glanz, Rimer, Viswanath, 2008), adapted to the healthy lifestyle behaviours for Dutch-Moroccan women in the Netherlands and adapted with the concepts of transnationalism, and intersectionality

To analyse the negotiation processes on meaning at different levels, the individual and the social in women’s perceptions, we use this model which explains health related behaviours as predicted by four perceptions: the pillars for behaviour change [17], the perceived seriousness of the problem, receptivity to poor-health, the profits and the barriers. This model has been elaborated to include recommendations for actions, motivating factors and self-efficacy in relation to health [18]. We expand the HBM model by embedding the women’s individual perceptions in their larger socio-cultural contexts, [19] from an intersectionality perspective by relating them to social identity markers, such as religion, culture, ethnicity, transnationality, aspects that are relevant for group formation in migrant groups [14]. By elaborating and especially by embedding the women’s perceptions within relevant social formations, we aim to understand feelings of group identity and identify negotiation processes of identity formation that are relevant for the women’s conceptualization of ‘health’ and ‘lifestyle’. This allows us to focus both on health beliefs and practices as well as on the social formations in which these are negotiated. Simultaneously, we aim to make the case that relating individual perceptions to the women’s social and cultural identities does not equal essentialism. Identity formation is a continuous process that occurs in daily interactions [20], it is not fixed but is ‘done’ within the women’s current contexts, in which lifestyle plays a central role (Fig. 1).

Our aim, to bring health and social/cultural factors together from the perspective of those whose lives are subject to the research, requires a method which allows individuals themselves to benefit from participating in the research. So we chose a research approach which offered the participants information and consultations about health and lifestyle with the researcher (KK), a dietician, after the group meetings and interviews were concluded. This led to the development of the following research question: what are the perceptions and experiences of health and lifestyle of older Moroccan-Dutch migrant women who, on average, have high rates of diabetes and hypertension. What does health mean to older Moroccan-Dutch women and what is their perception of a ‘healthy lifestyle’?

This qualitative research approach integrates research, education and consultation. The research period was divided into two phases between April and September 2015: in the first phase, in Amsterdam, seven group meetings were held (group discussions followed by education) on health, food, exercise and Ramadan (18 June-16 July 2015) with a varying number of participants [3–16]. The yearly Ramadan offers participants the possibility to reflect on health and lifestyle. Therefore, in the second phase of the study, during Ramadan and after, twelve individual in-depth interviews were conducted. One participant-observation day was held during the preparations and celebration of a Ramadan *iftar* (breaking a fast day). The woman who made the participation day possible, was an elder member of the group meetings in Amsterdam. She was known and regarded as an ‘informal leader’ by the members of the group who regularly asked her for advice. This method offered the women the opportunity to put their own themes on the agenda of the group meetings which, in turn, enabled the researcher to provide tailor-made information based on the questions and needs that the women raised. Individual interviews helped to deepen the insight into the women’s specific needs and perceptions. The women were eager to pose their questions openly to the researchers, Dutch women without a migration background, who specialized in gender, health, diversity and dietary requirements.

Participants for the group meetings were recruited from two migrant women organizations in Amsterdam. These women formed ‘natural groups’ as they met regularly. In natural groups the interaction between participants is maximised providing the most accurate insights into a shared group culture [21]. During these group discussions, women who met the inclusion criteria (female, first-generation migrant, aged 40–80 years and sufficiently competent in the Dutch language), were asked to take part in an individual in-depth interview and an observation day (see Table 1). Seven respondents living in Amsterdam were willing to participate. Snowball sampling was applied; six women participated from smaller towns in the centre and south of the Netherlands [22]. Purposive sampling was used to get an optimal variation of interviewees, all first generation, in age, ethnicity (Tamazight/Berber or Arabic speaking) and region of origin [23]. Most of the Moroccan-Dutch women (80%) come from the north of Morocco [24]. A high number of older Moroccan-Dutch women are illiterate, so our goal was to include women who had received both low and intermediate level education in order to increase the richness and validity of the data (see Table 1).

**Table 1:** Inclusion criteria for women, Phase two: Interviews

Moroccan-Dutch woman	
First generation migrant	
40–80 years	
Adequate level of Dutch	

A total of 57 women participated. In Phase 1 (the ‘natural’ group meetings) 44 women participated, 38 of which were first-generation, born in Morocco, and 6 who were younger, second generation, born in the Netherlands, aged between 22 and 69 years and living in Amsterdam. Of these 44 women, 26 were married, 9 divorced, 3 widowed; all 6 of the second-generation women were single. All first-generation women had given birth to 1–9 children and most of the women had children still living at home. All the women were responsible for the household and one had a paid job. Most first-generation women were illiterate and spoke limited Dutch. All second-generation women were studying (see Table 2).

**Table 2:** Number of participants distributed by different forms of data

Data collection	7 group discussions and training sessions Phase 1	12 individual in-depth interviews Phase 2	1 observational day Phase 2
Total no. participating women	*N* = 38 first generation	*N* = 12 first generation	*N* = 1 first generation
	*N* = 6 s generation		
*N* = 57	Total *N* = 44	Total *N* = 12	Total *N* = 1

The women were interested in knowing their weight and height so, before the discussions and interviews were held, they were weighed, their heights were estimated and their BMI was calculated. According to the BMI classifications, 3 of the first generation women were of a healthy weight, while the other women were overweight, obese or severely obese. Of the second-generation women, 2 women were overweight and 4 were of a healthy weight.

All women in Phase 2 (*n* = 12) and the woman who took part in the observation day (n = 1) were first-generation, aged 40 to 66 years. Seven women had migrated to the Netherlands during the 1980s as brides or because of family reunion, and the other six came as children. Five women had received no formal education. The other women had finished primary school, 2 had attended higher education in the Netherlands, and 4 had part-time jobs. Six women were overweight, 3 were obese and 1 was severely obese. Two of the women who had attended higher education women were not weighed.

Initial topic lists were composed before the group meetings and in-depth interviews were held. To create a good atmosphere and to build rapport, a number of less sensitive subjects such as health and healthy diet were discussed at first before more sensitive subjects like fasting were broached. The aim was to recruit 30–35 participants, which is usually enough to achieve data saturation [25]. The discussions and interviews were held in Dutch. During the group meetings some of the women translated for their peers and several languages were used: Darija (Moroccan Arabic), Tashelhiyt (the Berber language of South Morocco) and Tarifit (the Rif language of North Morocco). The women knew each other and were friends, and informal translations were ‘natural’.

Member checks were carried out of all the discussions, training sessions and interviews throughout the data collection period [26]. After every subject and every session, for example, the researcher verified whether the summary was an accurate interpretation. Data triangulation (group discussions, in-depth interviews and observations) was also carried out to enhance the validity of the research results [27]. The group discussions and interviews were audio recorded and transcribed verbatim.

Before each group discussion and training session began, all the women taking part were informed about the study. Written consent was given by some women but not all. Many of this latter group of women had rather low levels of literacy, and refused or could not give written consent, but did give oral permission. At the end of the information session about the nature of the research, it was made clear to the group that they were free to leave. The tape recording was then started; none of the women left but actually came specifically to join the discussions and training sessions. In the Netherlands, ethical approval is not required for this type of study under the Medical Research involving Human Subjects Act (http://www.ccmo.nl/nl).

The results of the research were fed back to the participants in two extra meetings held in 2016 and 2017 and the women stated that they agreed with their contents. This agreement serves as an extra check on the reliability of the results and analysis [28].^1^

Thematic content analysis was applied to analyse the data [29]. After six group discussions and two interviews, based on open coding, a thematic framework was developed by clustering and combining (‘thematic coding’). This framework was modified after the last group discussion and the fifth interview in the style of a grounded theory approach [21]. All the rest of the collected data, including the remaining interviews, were placed into the developed framework to complete the analysis and contribute to theoretical saturation [29]. All phases during data analysis were discussed and evaluated within the research team (researcher triangulation) which increased the validity of the study [30].

## Results

### Perceptions on health and lifestyle

What does health mean to older Moroccan-Dutch women and what do they perceive of as a ‘healthy lifestyle’? First, we explore how women take an integrated approach to health which incorporates both physical aspects as well as more spiritual beliefs. Overall, health is a central theme for Moroccan women, and they also feel responsible for their families’ health. Although health was defined mostly in a ‘functional capacity’, “*That you can walk, sport, move*”, the negative definition was stated as well: “*the absence of illness”.* One woman expressed a moral position i.e. health was “*something to be grateful for, but you must be active to be healthy and it requires commitment.”* Most women focused on health as a physical condition of body and mind and as a ‘given’, as constitutional and genetic: “*In your body and in your family*.” Finally, some women spoke about health as being God given, “*min Allah*”, and as determined by God, “*maktoub min Allah*.” This is not as fatalistic as it sounds because “*Your body is a gift from Allah and you should take care of it in the best way you can.”* This combined acceptance of the human physical condition with the possibility of controlling one’s health by one’s own actions does not focus on the individual as being entirely responsible for her or his own health: “*Finally it is God who decides.”*

Thus, while perceiving health in terms of a dominant, more individualised Dutch discourse, the women’s perspectives on lifestyle seem highly embedded in a cultural, religious, aged and gendered context, which we explored at greater depth in five major themes. In the first theme: *Generations and lifestyle* the women share their perceptions on the different lifestyles between generations. In the second: *Being a woman and an aging mother,* gender roles in relation to health are placed in a cultural context*.* The third theme: *Disease and treatment* focuses on being Muslim migrants in Dutch society. In the fourth theme the relationship between health and lifestyle is perceived from the women’s *Religious and cultural perspectives*. Finally in the fifth theme: *Transnationalism,* the relationship with Morocco was described as the main characteristic of daily life in which health beliefs and perceptions played a role.

### Generations and lifestyle

All the women knew someone who was dieting and many women had tried to lose weight before. They explained that they talked a lot about healthy food. The women discussed how the first-generation ate as they were taught in their youth in Morocco: a lot of olive-oil, white bread and large portions. These habits were difficult to change and were embedded in religious beliefs: “*That is what the prophet ate as well*.” The amount of olive oil consumed during breakfast and the quantity of food consumed during meals seemed particularly noteworthy for many women, with one woman calculating that a Moroccan family consumed about 40 l of olive oil a year, which they believed to be healthy. Often, families ate together from large joint plates and, especially in the evening at dinner time, they ate a lot. An individual plate might consist of half meat, about one third potatoes or other starch product and a small portion of ‘over-cooked’ vegetables. They ate (mostly white) bread with every meal, used as a utensil to take food and, with bread being seen as “*a special gift from Allah”*; it should not be thrown away. The use of sugar versus sweeteners was often discussed during the meetings. Although most women tried to consume less sugar in Moroccan mint tea, eating Moroccan biscuits was a daily food habit for most women.

The second generation (who participated in the natural groups) explained that they had developed a more ‘Dutch’ view of health and lifestyle. They did not appreciate Moroccan traditional dishes because of the amounts of fat/olive-oil, while preferring what they considered to be more ‘Dutch’ dishes, such as pizza and noodles. For some mothers this preference was perceived as a rejection of their care. Daily exercise was practiced in different ways for the first and second generation: the first generation walked more often, sometimes in small groups of friends or relatives, whereas the second-generation went to the gym. Two women mentioned they walked outside because it was without costs: “*I have to lose weight because my general practitioner told me I have a risk of developing diabetes. It is in my family. That’s why so many Moroccan women are walking now. They fear diabetes and it is cheap.”*

### Being a women and an aging mother

As a woman and a mother the women were responsible for housekeeping and the preparation of food. Most group participants had large families with three to nine children and more than half of the women still had one or two almost grown up children living at home. Although all the women were responsible for housekeeping, family members, especially husbands assisted them, for instance with shopping. Some women spoke about how marriage and being a mother could lead to weight gain. After childbirth in particular, other women would cook many delightful dishes and present them to the new mother. Consequently, marriage and motherhood created a specific state of mind: “*Moroccan women, after marriage and their first child, they become fat. They say: ‘I am married and I have children. I am done.’ For me as well: in two years, I gained 20 kilos.”*

Ageing and being a mother of grown-up adults made women reflect on health and healthy lifestyle and on the priorities in life: *“In the past I told my mother, ‘Please, mother, you are too overweight, it’s not healthy’. My mother’s answer: ‘I would rather die with a full stomach, than pass away with an empty one*.’” Motherhood is the main goal for most women and as mothers, they assumed, they would always be primarily responsible for all the problems concerning their young or grown-up children. But daily care is over and ageing requires reconsidering the meaning of life: “*At this age, a Moroccan woman’s life’s work is over. Her daily care for her children is gone, but she feels accountable for the fact that these children are who they are now. Some women suffer because of their children, and at the same time they have no more prospects in life*.” “*Even if their children are successful, they come at the weekend and that’s it.”*

Aging prompts the women to reflect and change. Lifestyle perceptions change when an ageing Moroccan-Dutch woman becomes ill or perceives a health threat such as diabetes. In such anxiety-provoking times, lifestyle perceptions seem to change and become more individualised. For instance (more) walking is mentioned as an individual responsibility in regard to being overweight and/or developing diabetes: *“when it runs in the family”*. They trust the GP and try to follow their advice, but limiting the amount of food, biscuits and sugar remains difficult.

### Disease and treatment

Half of the women said that they did not feel healthy and talked about hypertension, muscle pain, diabetes, constipation and vitamin D insufficiency (for which most women took supplements). And although all the women were well aware of diabetes as a major health problem among Moroccans: “*This disease is today’s fashion*”, not all of them acknowledged the risk of developing such lifestyle-related diseases. One woman claimed she developed diabetes because of the stress she had endured during the Hadj (pilgrimage to Mecca). Also, the notion of multi-morbidity was unknown.

Practically all the women indicated that stress was their most important health problem “*That is what you hear from all of us; every woman complains about stress*”, which they attributed to a variety of reasons: [1] their children and the responsibility they felt for their children’s future prospects [2]; their husbands who restricted their freedom, and [3]; the influences of social control and the pressure to keep your good reputation by behaving well, offering hospitality and indulging visitors - “*The outside control*” refers to gossip, or “*What people say about you*.” One project manager of the women’s organization explained how illiterate Dutch-Moroccan women often find planning their daily schedule difficult, and develop stress because of the lack of time to finish their tasks. In the relaxed atmosphere of trust during the group meetings, talking about causes and solutions, stress was however mentioned as a taboo. Most women had their own coping mechanisms, such as discussing their problems with their general practitioner or a physiotherapist. However, the GP had limited time per consultation, physiotherapy was limited to twelve sessions, and further therapy was unaffordable. The women often combined regular therapies with traditional and religious healing methods such as washing with texts from the Qur’an in Morocco and *hijama* or cupping, even if they had to pay for it. One woman had just successfully passed her exams to be a *hijama* therapist: “*It helps for most of your problems and the prophet used it himself*.”

Some of the women planned to have traditional therapy to treat their chronic pain when they were in Morocco during the holidays. “*Sitting in hot Sahara sand”* was an activity familiar to several women. In the Netherlands, the women mentioned using herbs and seeds for pain relief and washing with water which had Qur’an texts dissolved in it was also used. According to most women, these practices were effective in reducing their symptoms. Some women commented that sometimes *sihr* (sorcery) and *ain* (the evil eye) are seen as a cause of disease, but these topics were taboo issues to discuss with Moroccans because of fear and with Dutch health care professionals because they just “don’t understand”.

### Religious and cultural perspectives

For most women, Islam is the main reference point in their lives, playing a role always and everywhere, and they prayed and fulfilled their duty to fast yearly during Ramadan. Preparations for fasting during Ramadan began weeks before it started so they could fast optimally. The kitchen was cleaned and special dishes and biscuits were prepared for the *iftar*, the evening meal which breaks the fast which was usually eaten in a family setting. Ramadan meant a time for prayer, for reflections on ones relationship with Allah, on one’s place in the world and how one could be a good and understanding person; it is a time for purifying mind, body and soul. Therefore, experiencing Ramadan puts people in a special state of mind, one which was considered to be very healthy: “*Ramadan is a holiday for the stomach*.” For most women the first days were difficult, but after a few days their bodies got used to it. Some women complained about gaining weight during these weeks, but most felt much better than ‘normal’ and lost two to five kilos, which would be regained in the months after Ramadan.

Advice from medical practitioners is usually against fasting in case of disease. For women with diabetes, for instance, fasting or not was a dilemma; they understood that they shouldn’t fast to protect their health, but felt impelled to do so as a religious requirement: “*Not fasting feels like failure. Not fasting for them means being weak in character, but Islam is orientated to health … … You should not fast in case of sickness. People think you should do so, but a lot has been taught to us incorrectly about Islam.”*

About half of the interviewed women celebrated Aid el Fitr, the feast at the end of Ramadan, during their holidays with family in Morocco, The Dutch translation of Ramadan as ‘sugar feast’, was considered inaccurate: “*It doesn’t mean that you should consume a lot of sweets. It is the feast of closing a religious phase in which you can receive religious earnings (azj) and blessings (baraka).*”

Hospitality is an important value, not only during Ramadan, but throughout the whole year: “*That is part of our culture, to nurture and indulge our guests*.” Delicious foods are prepared for all guests, and guests are considered impolite if they refuse or do not to eat a certain amount of these foods. Throwing away leftover foods is simply not part of the cultural tradition and, in the same way as not throwing bread away, it is against Islam.

### Transnationality

To stay connected with family in Morocco, the women use their mobile phones or Skype. During holidays they visit relatives, sometimes several times a year. All the interviewed women watched Moroccan television daily, with television and internet providing information about health and lifestyle. All the women had heard of Dr. Mohammed El Faid and Dr. Nabil El Ayachi who teach about healthy food and lifestyle issues, and all were familiar with at least one of these ‘public influencers’. They commented that they enjoyed watching them, and mentioned putting some of their recommendations into practice and discussing these suggestions with others. Moreover, a noticeable lifestyle change among women living in Morocco, mainly in cities, was observed by many interviewed women: women were walking outside in large groups to exercise and more women were going to gyms. Overweight and obesity were being perceived as less attractive; a change in the beauty ideal was recognised, from ‘voluptuous’ women or ‘fat’ women to ‘thin’ or ‘normal’ sized women, indicating that ‘fat’ was no longer ‘normal’. This was preferred by both women and men: “*In Morocco, you should go to the beaches and the parks: you will see all kinds of women walking, running, maybe more than here. Even men, they don’t like fat women anymore. They prefer thin women*.”

## Discussion

What does health mean to older Moroccan-Dutch women and how do they perceive a ‘healthy lifestyle’? This study shows that health is important for Moroccan women and that they also feel responsible for their families’ health. While they perceive health in more or less the same terms as those of the dominant Dutch discourse, i.e. individually related to aging and constitution, their view on the ultimate responsibility for health is different. Finally health is seen as a gift, from God. Moreover their perspectives on lifestyle seem not focused on individual responsibility or as something they can control individually, as is the main perception of Dutch healthcare. Lifestyle for them is rather highly embedded in their cultural, religious, aged and gendered context.

**Fig. 2:**
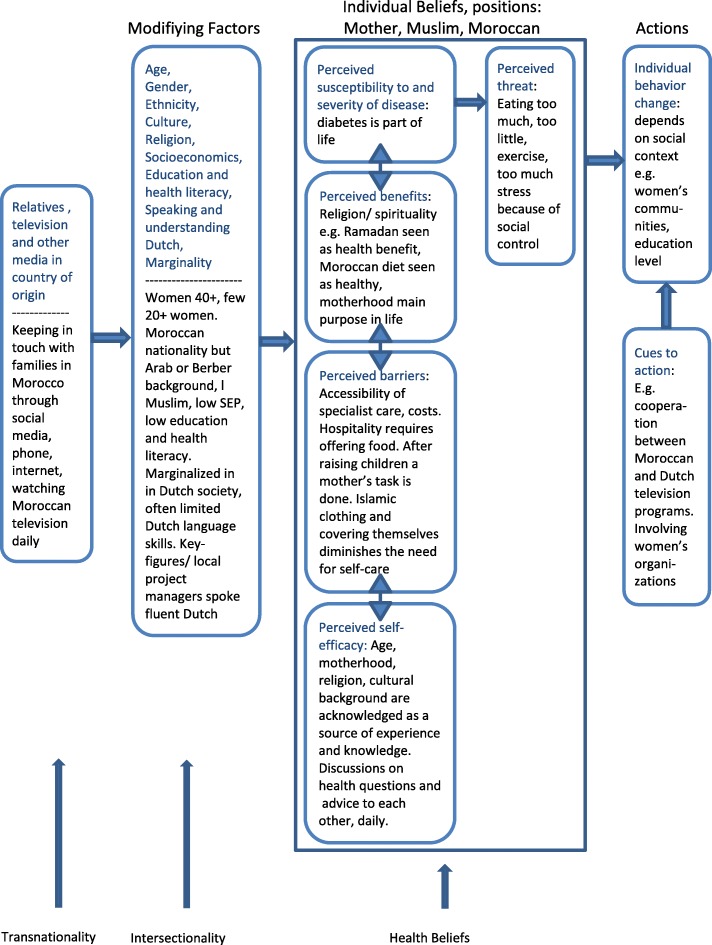
Completed Conceptual Schedule

We started with the Health Belief Model (HBM), i.e. individual beliefs and perceptions, and expanded it by embedding the women’s individual perceptions in their larger cultural and social contexts (an intersectionality framework). When applying the HBM to our findings, we can identify several barriers that the women themselves recognised and discussed (Fig. 2). First, a barrier relating to the physical condition and genetic vulnerability within families, for instance, whether diabetes runs in the family. A second barrier is perceived in access to health care created by differences in language and religious and cultural beliefs. A third barrier can come from supernatural powers, such as *sihr* (sorcery) and *ain* (the evil eye) [31]. These issues are often taboo and difficult to discuss, and are poorly addressed in their community or in health programmes, if they are mentioned at all. The fourth type of barrier is embedded in lifestyle, such as the high value attached to hospitality and the food-related indulgence shown at life events such as a new birth, or visiting family *iftars* during Ramadan, and participating in the yearly Islamic feasts. Most women reported lifestyle changes as being difficult to achieve and this was accepted as a given.

As regards the perceived benefits, the women stated that exercising or doing sport, and in particular walking outside, is cheap and beneficial for everyone, but that limiting food portions was more difficult. Social benefits or ‘earnings’ in social interaction, such as a good reputation in the community by offering hospitality and indulging people, prevented gossip and social control, which seemed to outweigh any individual health benefits.

Lifestyle is clearly related to social phenomena [6, 32, 13]. The themes identified were linked directly to the identity constructions Moroccan, Muslim and mother. In *Disease and treatment*, for instance, it becomes clear that treatment is not only perceived as bio-medical care. Health and lifestyle perceptions arise from views on medical treatment and are related to, and supportive of, the women’s Moroccan and Islamic identity, such as practising traditional healing, ‘prophet medicine’ like *hijama* or cupping, or using Qur’an texts, measures not taken by most people in the Dutch majority, and claimed as Moroccan and Islamic. *Transnationality* showed how belonging to two nations [33] provides a means of supporting Moroccan identity for Moroccan-Dutch and of practicing these forms of healing.

The foregone shows how the women experienced their realities as grounded in several categories of difference and their intersections. The women prioritise their main identities as Muslim, mother and Moroccan in the Netherlands, and these identities reinforce each other in relation to lifestyle and perceptions of health. Besides age, gender, religion and ethnicity, other modifying factors, such as income, speaking Dutch etc., are hardly referred to by the women.

Our study confirms other studies which have illustrated that lifestyle perceptions change when an ageing Moroccan-Dutch woman becomes ill or perceives a health threat, like diabetes [7]. With regard to women’s perceptions of susceptibility to disease and the experience of severe illness, this did indeed motivate them to change behaviours for instance by exercising/walking more. Our findings also suggest that the advice of a GP, an authority figure, may play a more important role in cues to action.

The women explain health and lifestyle not only within their context, but in relation to their life course too. As the women age and children grow up, their role as a mother changes, not only into becoming a mother of older children, but a mother of children who have become rather ‘Dutch’. When adult children leave the house, the woman feels her purpose in life has been completed and perceives a loss of prospects, sometimes feeling rejected. Nevertheless, they still feel and are seen as responsible for their children’s well-being, while simultaneously losing the authority they had had as a mother. *Transnationality* showed how the relationship with the country of origin was continued and how lifestyle is socially embedded. *Transnationality* is also expressed in how changes in Morocco are discussed and reflected on in the Netherlands, how identities are not fixed, and how the women notice that perceptions of health and lifestyle are changing both in the Netherlands and in Morocco.

## Limitations and strengths

There are some limitations to the study. First, the group discussions may have inhibited some women from giving their genuine answers, as the Moroccan community is known for strong social control [34]. However, the use of ‘natural’ groups also helped to counter this problem. Secondly, more observation days than one had been planned, but data collection fell during summer holidays, which hampered recruitment. Many women went on holiday to Morocco for a couple of weeks. Nevertheless, the total number of participants sufficed, and is even high for a group which is generally difficult to reach [35]. The ‘natural groups’ and group discussions supported recruitment through snowball sampling. In line with this, a strength of this study is that data triangulation was achieved by collecting different forms of data sources, and through member checks. Besides the recruitment issues raised during Ramadan, this time frame also offered particular details about health behaviours in relation to religion, and hence elicited more in-depth religious perceptions on health, which is a strength of the study.

The fact that the interviewer (KK) was a ‘white’ Dutch non-religious female interviewer may have influenced the results. Women might have been more open to someone who could understand their religion and had the same background. On the other hand, this may also have contributed to feelings of safety among the participants because the interviewer had no connection to their communities. The fact that the researcher (KK) was familiar with Morocco did create trust and confidence among the respondents.

## Recommendations

In the Netherlands lifestyle interventions are implemented to ameliorate the health of vulnerable groups. Interventions usually take place at an individual level. But as this research shows: lifestyle can also be seen as a social phenomenon and related to central identity formations. Interventions should also be understood from the perspective of what lifestyle means for people’s social identities and thus, offered from a social rather than an individual perspective. Women’s organizations, for instance for migrant women, can play an important role in healthier lifestyle developments. Discussions on health, food, exercise and social identities such as religion and motherhood, being a migrant women with family in the country of origin, can be crucial to open up new lifestyle options. This research offers three other recommendations to inform these interventions. First, adapt interventions to the women’s preferences and practices, and recognise Ramadan as a ‘window of opportunity’ to influence long-term lifestyle changes. During Ramadan all the women focus on health and food, often lose weight and change their lifestyles, as fasting strengthens their spiritual motivation to take good care of their families and of themselves. Also recognise the opportunity that the natural life stages present, such as pregnancy and childbirth, as these also offer a ‘window of opportunity’ because the women report gaining extra weight during these times.

Second, collaboration between health care professionals in the Netherlands and Morocco could be stimulated with joint interventions of Moroccan and Dutch health care services supplying information and education about healthy eating habits. It is well known that older Moroccan-Dutch women watch a lot of Moroccan television. So, television cooking shows featuring Moroccan master chefs, for instance, could improve traditional Moroccan dishes making them healthier, but still in line with the traditional ingredients and flavours. The Healthy Sisters, (https://healthysisters.nl/) two Moroccan second-generation sisters, who have a food and healthy website and blog, are such Moroccan-Dutch role-models, especially for the second generation. Older Moroccan women enjoy getting together for ‘cooking groups’, where they can cook and socialise. This is an ideal opportunity to learn and practice a healthy lifestyle, but specific role models for older Moroccan women are needed too.

The third practical recommendation is that more attention be devoted to the prevention of NCDs, especially diabetes which is a very common and well-known disease for older Moroccans, experienced as a ‘fact of life’ and unavoidable. People seem unaware of the severity of diabetes, and multi-morbidity requires serious attention [9]. Other projects showed that prevention can be achieved by group education through migrant women organizations and community centers [14].

## Conclusion

Our results point to a view of health and lifestyle for older Moroccan women that is a much broader phenomenon than food and exercise alone. The concepts are heavily embedded in the women’s main identities as Moroccan, Muslim and mother. In order to match the target group’s perspectives best, healthy lifestyle interventions should also focus on the social elements which are experienced as being most important by the women themselves.

## Footnotes

1 Initial findings of this study were announced in a professional Dutch journal [28].. The editors of this journal support an international publication.
